# Extent of interocular (a)symmetry based on the metabolomic profile of human aqueous humor

**DOI:** 10.3389/fmolb.2023.1166182

**Published:** 2023-03-29

**Authors:** Karolina Pietrowska, Diana Anna Dmuchowska, Adrian Godlewski, Emil Tomasz Grochowski, Malgorzata Wojnar, Wioleta Gosk, Joanna Konopinska, Adam Kretowski, Michal Ciborowski

**Affiliations:** ^1^ Clinical Research Center, Medical University of Bialystok, Bialystok, Poland; ^2^ Department of Ophthalmology, Medical University of Bialystok, Bialystok, Poland; ^3^ Department of Endocrinology, Diabetology and Internal Medicine, Medical University of Bialystok, Bialystok, Poland

**Keywords:** ophthalmology (MeSH), symmetry, aqueous humor (AH), mass spectrometry, LC-MS/MS, metabolomics (OMICS)

## Abstract

**Aims:** Interocular comparison of the metabolomic signature of aqueous humor (AH) was performed. The aim of the study was to quantitatively evaluate the symmetry in concentrations of various metabolites belonging to different categories.

**Methods:** The study included AH samples from 23 patients, 74.17 ± 11.52 years old, undergoing simultaneous bilateral cataract surgery at the Ophthalmology Department of the Medical University of Bialystok, Poland. Liquid chromatography coupled with tandem mass spectrometry (LC-MS/MS)-based targeted metabolomics and lipidomics analyses of AH samples were performed using the AbsoluteIDQ^®^ p180 kit. Out of 188 metabolites available in the kit, 67 were measured in the majority (>70%) of the samples: 21/21 amino acids, 10/22 biogenic amines, 9/40 acylcarnitines, 0/14 lysophosphatidylcholines, 21/76 phosphatidylcholines, 5/15 sphingolipids, and 1/1sum of hexoses.

**Results:** The comparison of both eyes revealed that the concentrations of metabolites did not differ significantly (*p* < 0.05) except for taurine (*p* = 0.037). There was moderate-to-strong positive interocular correlation (r > 0.5) between most metabolites regarding concentration. This was confirmed by the high intraclass correlation coefficient (ICC) values of different levels, which varied for the different metabolites. However, there were exceptions. Correlations were not significant for 2 acylcarnitines (tiglylcarnitine and decadienylcarnitine) and 3 glycerophospholipids (PC aa C32:3, PC aa C40:2, and PC aa C40:5).

**Conclusion:** With a few exceptions, a single eye was found to be representative of the fellow eye in terms of the concentration of most of the analyzed metabolites. The degree of intraindividual variability in the AH of fellow eyes differs for particular metabolites/metabolite categories.

## 1 Introduction

Although eyes are paired organs, numerous anatomic asymmetries have been described, even in ophthalmically healthy patients (Cameron et al., 2017). Asymmetry has been demonstrated, e.g., for intraocular pressure, axial length, corneal, optic nerve head, and retinal parameters (Li et al., 2013; Hwang et al., 2014; Jacobsen et al., 2015; Linderman et al., 2018; Baniasadi et al., 2020; Jiang et al., 2021; Albarrán-Diego et al., 2022; Song and Hwang, 2022).

Aqueous humor (AH) is produced by the ciliary body, a part of the uveal tract. Although no studies evaluating the interocular symmetry of the ciliary body are available, another constituent of the uveal tract, choroid, has been investigated extensively. Choroidal differences exist between normal fellow eyes in adults, i.e., in the absence of obvious pathology (Yang et al., 2016; Kim et al., 2021; Lu et al., 2022). Consequently, one may expect that the metabolomic profile of AH may differ between fellow eyes.

Metabolomics is the complex assessment of metabolites, small molecules, lipids, and carbohydrates, among others (Pietrowska et al., 2017; Pietrowska et al., 2018a). It facilitates knowledge about physiology and pathophysiology as well as biomarkers of occurrence, type and stage of disease, and response to treatment. It thus allows the identification of disturbed metabolic pathways. One of the advantages of metabolomics is its close relation with phenotype (Kell et al., 2005). The identification of sources of bias is of the utmost importance for proper research planning and the interpretation of results. Biological variability due to exogenic and endogenic factors is one such source of bias (Crews et al., 2009; Kim et al., 2014). It is therefore important to know whether clinically relevant differences between both eyes can be expected. Previous reports on the biological variability of other fluids, e.g., serum, plasma, urine, and cerebrospinal fluid are available (Crews et al., 2009; Kim et al., 2014). However, they are not transferable to AH due to differences in their composition. To date, there have been no reports on the within-subject variability of AH collected at the same time. Our study aims to address this gap.

Metabolomics is a young and dynamic discipline that is attracting growing attention in ophthalmic research. The AH metabolomic profiles are altered in ophthalmic and systemic diseases, e.g., glaucoma, pseudoexfoliation syndrome, diabetic eye disease, and myopia, among others (Pietrowska et al., 2018b; Buisset et al., 2019; Myer et al., 2020a; Myer et al., 2020b; Dmuchowska et al., 2020; Grochowski et al., 2020; Dmuchowska et al., 2021a). However, there are still ambiguities, including the issue of biological variability. Some studies have included one eye of an individual, selected randomly or according to disease severity. However, the laterality of the included eyes may have affected the results. Consequently, the question arises whether one eye is representative for the other eye of the same patient in terms of AH metabolomics signatures. The detection of asymmetry may support proper study planning and interpretation. Furthermore, it might facilitate personalized medicine and indicate the need for further assessment of potential local factors (e.g., blood supply) that may play a significant role and would need to be taken into account in future studies.

The aim of the study is to quantitatively evaluate the symmetry of concentrations of various metabolites belonging to different categories. This targeted metabolomics and lipidomics study compares the AH composition between fellow eyes of the same patients. To the best of our knowledge, the current investigation presents the first report on interocular comparison of the metabolomic signature of AH.

## 2 Materials and methods

### 2.1 Study participants and sample collection

The study included AH samples from 23 patients undergoing simultaneous bilateral cataract surgery (SBCS) at the Ophthalmology Department of the Medical University of Bialystok, Poland, from 21 January 2021 to 02 December 2021 (Dmuchowska et al., 2021b). The systemic comorbidities and medications used are presented in Supplementary Table S1. The following were criteria for exclusion from the study: the presence of concomitant ocular disorders except for cataract, history of surgery or trauma, and/or diabetes mellitus.

All patients underwent a standard preoperative procedure. At 20 min before the surgery, they simultaneously and bilaterally received topical application of the following: proxymetacaine hydrochloride, tropicamide, phenylephrine, levofloxacin, diclofenac, and timolol. One hour before the surgery, the patients received oral hydroxyzine for mild sedation. Standard disinfection with 5% ophthalmic povidone iodine was implemented 2 min before beginning the procedure. Before cataract extraction, the anterior chamber of the eye was punctured using a 30 G needle; approximately 50–100 μL of AH was aspirated, transferred to Eppendorf tubes (Eppendorf, Hamburg, Germany), frozen, and stored at −80°C until the analysis. The surgeries were performed in the morning from 8 a.m. to 12 p.m., with a timespan of within 15–20 min between fellow eyes of the same patients.

### 2.2 Metabolomics analysis

The targeted metabolomics analysis of AH samples was performed using liquid chromatography coupled with tandem mass spectrometry (6470 LC–MS/MS, Agilent Technologies, Santa Clara, California, United States), applying the methodology and reagents included in the AbsoluteIDQ^®^ p180 kit (Biocrates Life Sciences AG, Innsbruck, Austria). This commercially available kit allows the quantitative measurement of 188 metabolites: 21 amino acids, 22 biogenic amines, 40 acylcarnitines, 14 lysophosphatidylcholines, 76 phosphatidylcholines, 15 sphingolipids, and the sum of hexoses. The sample preparation was performed according to the Kit User Manual, which has already been described in the literature (Sawicka-Smiarowska et al., 2021), with minor modifications. The sample volume used for the analysis was optimized (unpublished data, 2022), and 30 µL of AH sample was used, instead of 10 µL recommended by the manufacturer for plasma/serum.

Spectral data processing and quantification were conducted using MetIDQ software (version Oxygen DB110-3005-290, Biocrates, Life Science AG, Innsbruck, Austria). The performance of the analytical assay was evaluated by analyzing quality control (QC) samples at three concentration levels, where the middle level (QC2) was injected three times. After normalizing the data based on the QC samples, metabolites with a coefficient of variation (CV) higher than 30% in the QC samples were excluded. Primarily, the data below the limit of detection (LOD) were treated as missing. Additional filtering was performed to retain metabolites detected in at least 70% of the samples. Subsequently, missing values were replaced with concentrations obtained based on the calibration curves but located below the lowest concentration point on the calibration curve. All obtained concentrations were divided by three to take into account higher sample volume used. Finally, a data matrix consisting of concentrations of 67 metabolites was forwarded for statistical analysis.

### 2.3 Statistical analysis

Statistical analysis was carried out using R software, version 4.0.5. For each metabolite, calculations of Spearman’s correlation coefficient and for paired Wilcoxon test between concentrations for both eyes were conducted. Chan YH has provided the following suggestion for interpreting Spearman’s correlation coefficient (r rho): >0.5 or ≤ 0.5, moderate to strong; −0.3 to 0.3, poor; in between, fair correlation (Chan, 2003). For the Wilcoxon test, both non-adjusted and adjusted *p*-values are presented (based on the Benjamini–Hochberg correction for multiple comparisons). The interclass correlation coefficient (ICC) was calculated for each metabolite between two eyes, assuming a two-way model and absolute agreement. Koo and Li have provided the following suggestion for interpreting ICC: < 0.50, poor; 0.50–0.75, moderate; 0.75–0.90, good; and >0.90, excellent (Koo and Li, 2016). In addition, the relative mean difference was calculated as left eye minus right eye divided by the average concentration in two eyes. Linear regression analysis was used to verify the relationship between certain metabolites and clinical parameters (age, sex, body mass index (BMI), and axial length (AXL)). All calculations were based on a significance level of 0.05.

SIMCA−P + 13.0.3.0 (Umetrics, Umeå, Sweden) was used for multivariate statistical analysis. It was not possible to build appropriate quality orthogonal partial least squares-discriminant analysis (OPLS-DA) models discriminating left eye from right one based on their metabolic profiles.

## 3 Results

The demographic and clinical data are presented in Table 1. As the metabolomic signatures of both eyes were analyzed from the same patients, the demographic and systemic factors remained the same. There was no statistical difference in axial length (*p* = 0.818) between fellow eyes.

**TABLE 1:** Baseline patient characteristics.

Characteristics	Value
Number of patients	23
Age, years, mean ± SD (years)	74.17 ± 11.52
Sex, female, n (%)	13 (56.5)
BMI, mean ± SD	27.99 ± 5.45
AXL, right eye, mm, median (Q1; Q3)	23.07 (22.56; 23.88)
AXL, left eye, mm, median (Q1; Q3)	23.01 (22.49; 23.82)

BMI, body mass index; AXL, axial length.

Overall, 67 of 188 metabolites fulfilled the criteria for inclusion based on the cutoff levels of CV and LOD: 21/21 amino acids, 10/22 biogenic amines, 9/40 acylcarnitines, 0/14 lysophosphatidylcholines, 21/76 phosphatidylcholines, 5/15 sphingolipids, and 1/1 sum of hexoses.

Table 2 presents the mean concentration values and relative mean difference between both eyes.

**TABLE 2:** Metabolite concentration values in fellow eyes.

Metabolite category	Metabolite name^a^	Eyes with metabolite concentration above LOD (%)	Mean concentration±SD in right eye [µM]	Mean concentration±SD in left eye [µM]	Relative mean difference (%) between fellow eyes	Coefficient of variation for QC2
Acylcarnitines	C0	100.0	17.009 ± 5.352	17.149 ± 5.226	0.8	7.3
	C2	100.0	4.208 ± 1.266	4.174 ± 1.193	−0.8	9.8
	C3	100.0	0.432 ± 0.246	0.448 ± 0.240	3.6	6.03
	C3-DC (C4-OH)	84.8	0.021 ± 0.007	0.021 ± 0.007	0.8	7.9
	C4	100.0	0.129 ± 0.041	0.136 ± 0.044	5.9	7.9
	C5	100.0	0.143 ± 0.048	0.150 ± 0.051	4.7	7.7
	C5-OH (C3-DC-M)	97.8	0.031 ± 0.007	0.032 ± 0.010	4.5	9.9
	C5:1	89.1	0.015 ± 0.010	0.015 ± 0.009	−3.0	13.4
	C10:2	89.1	0.031 ± 0.009	0.029 ± 0.006	−6.3	27.0
Aminoacids	Ala	100.0	405.507 ± 117.704	425.304 ± 124.295	4.8	4.4
	Arg	100.0	108.304 ± 27.027	111.536 ± 26.590	2.9	10.2
	Asn	100.0	36.275 ± 6.141	37.268 ± 6.595	2.7	3.5
	Asp	100.0	3.153 ± 1.461	3.806 ± 2.040	18.8	2.2
	Cit	100.0	4.942 ± 1.723	4.996 ± 1.803	1.1	4.3
	Gln	100.0	830.638 ± 106.162	848.333 ± 124.914	2.1	7.5
	Glu	100.0	10.390 ± 4.624	11.655 ± 5.516	11.5	6.02
	Gly	100.0	35.270 ± 18.793	37.725 ± 20.386	6.7	8.2
	His	100.0	76.246 ± 13.773	78.449 ± 12.471	2.9	9.7
	Ile	100.0	70.725 ± 22.952	73.319 ± 23.170	3.6	9.3
	Leu	100.0	161.957 ± 38.741	167.768 ± 36.503	3.5	8.9
	Lys	100.0	177.449 ± 26.257	186.232 ± 21.099	4.8	9.9
	Met	100.0	35.342 ± 5.680	36.412 ± 5.666	3.0	8.9
	Orn	100.0	27.881 ± 8.664	28.735 ± 8.760	3.0	10.3
	Phe	100.0	108.609 ± 19.193	112.507 ± 16.839	3.5	8.4
	Pro	100.0	50.878 ± 23.456	53.620 ± 26.263	5.3	7.2
	Ser	100.0	201.681 ± 46.241	204.536 ± 38.421	1.4	8.4
	Thr	100.0	125.116 ± 27.449	127.826 ± 28.299	2.1	5.0
	Trp	100.0	30.839 ± 4.088	31.946 ± 5.184	3.5	9.1
	Tyr	100.0	96.000 ± 12.242	99.913 ± 14.891	4.0	9.4
	Val	100.0	275.348 ± 59.049	285.710 ± 52.170	3.7	10.0
Biogenic Amines	ADMA	100.0	0.539 ± 0.114	0.548 ± 0.113	1.6	5.6
	alpha-AAA	100.0	1.814 ± 0.579	1.705 ± 0.542	−6.2	12.2
	Carnosine	100.0	0.026 ± 0.033	0.027 ± 0.030	4.3	10.5
	Creatinine	100.0	53.261 ± 18.331	53.604 ± 19.004	0.6	3.8
	Kynurenine	100.0	0.948 ± 0.198	0.973 ± 0.241	2.6	8.9
	Met-SO	100.0	0.533 ± 0.164	0.535 ± 0.176	0.3	11.02
	Putrescine	100.0	0.155 ± 0.051	0.160 ± 0.047	3.5	5.6
	SDMA	100.0	0.501 ± 0.158	0.507 ± 0.171	1.3	6.5
	t4-OH-Pro	100.0	5.038 ± 3.050	5.207 ± 2.820	3.3	8.1
	Taurine	100.0	65.652 ± 25.612	75.319 ± 29.669	13.7	5.9
Glycerophospholipids	PC aa C32:0	97.8	0.036 ± 0.028	0.038 ± 0.044	6.7	6.4
	PC aa C32:1	80.4	0.021 ± 0.016	0.021 ± 0.021	1.5	8.5
	PC aa C32:3	71.7	0.001 ± 0.001	0.001 ± 0.001	13.1	18.2
	PC aa C34:1	100.0	0.264 ± 0.209	0.258 ± 0.302	−2.2	7.03
	PC aa C34:2	87.0	0.135 ± 0.118	0.124 ± 0.158	−8.7	8.03
	PC aa C36:1	100.0	0.068 ± 0.054	0.067 ± 0.078	−1.2	9.8
	PC aa C36:3	76.1	0.058 ± 0.053	0.054 ± 0.076	−6.9	8.02
	PC aa C36:4	95.7	0.086 ± 0.080	0.089 ± 0.126	2.6	9.1
	PC aa C38:3	89.1	0.032 ± 0.033	0.030 ± 0.041	−5.4	7.0
	PC aa C38:4	73.9	0.077 ± 0.079	0.075 ± 0.102	−3.7	8.7
	PC aa C38:5	73.9	0.030 ± 0.029	0.028 ± 0.042	−6.4	8.5
	PC aa C38:6	73.9	0.036 ± 0.037	0.036 ± 0.051	−1.4	7.3
	PC aa C40:2	76.1	0.001 ± 0.001	0.001 ± 0.001	23.1	5.7
	PC aa C40:5	76.1	0.007 ± 0.007	0.007 ± 0.009	3.6	10.03
	PC aa C40:6	82.6	0.032 ± 0.022	0.032 ± 0.035	0.1	8.0
	PC ae C34:1	97.8	0.013 ± 0.014	0.013 ± 0.018	0.1	8.2
	PC ae C36:3	89.1	0.005 ± 0.006	0.006 ± 0.010	3.4	7.9
	PC ae C36:4	73.9	0.021 ± 0.018	0.019 ± 0.022	−11.1	6.4
	PC ae C36:5	71.7	0.010 ± 0.011	0.010 ± 0.018	2.1	9.7
	PC ae C38:4	76.1	0.012 ± 0.014	0.010 ± 0.020	−13.2	8.3
	PC ae C40:5	91.3	0.003 ± 0.003	0.003 ± 0.004	23.2	7.0
Sphingolipids	SM C16:0	73.9	0.146 ± 0.148	0.143 ± 0.214	−2.4	9.9
	SM C16:1	97.8	0.025 ± 0.019	0.024 ± 0.026	−1.8	10.2
	SM C18:0	71.7	0.053 ± 0.051	0.055 ± 0.079	4.5	10.6
	SM C18:1	95.7	0.032 ± 0.032	0.030 ± 0.034	−8.5	9.9
	SM C24:1	71.7	0.076 ± 0.088	0.078 ± 0.130	2.5	12.6
Sugars	H1	100.0	4,045.594 ± 2,514.684	3,940.290 ± 2,260.815	−2.6	9.8

^a^ The full list of individual metabolites is available at https://biocrates.com/wp-content/uploads/2022/02/biocrates-p180-list-of-metabolites-v2-2021.pdf (accessed 30 October 2022).

LOD, limit of detection; QC, quality control.

After the application of Benjamini–Hochberg correction, the comparison of both eyes with Wilcoxon tests revealed that the concentrations of metabolites, except for taurine, did not differ significantly (Table 3). In general, there was a moderate-to-strong positive interocular correlation (r > 0.5) for most of the metabolites. This was confirmed with ICC values of different levels, which were variable throughout different metabolites. However, there were exceptions: correlations were not significant for 2 acylcarnitines (C5:1 and C10:2) and 3 glycerophospholipids (PC aa C32:3, PC aa C40:2, and PC aa C40:5).

**TABLE 3:** Comparison of metabolite concentrations between fellow eyes.

Metabolite category	Metabolite name^a^	Eyes with metabolite concentration above LOD (%)	ICC between both eyes		Correlation between both eyes		*p*-value of WILCOXON test	
			ICC	95% CI	r	p^b^	Without correction for multiple comparisons	With BENJAMINI–HOCHBERG correction for multiple comparisons
Acylkarnitines	C0	100.0	0.928	0.838–0.969	0.93	**<0.001**	0.6	0.7
	C2	100.0	0.927	0.837–0.969	0.87	**<0.001**	0.8	0.9
	C3	100.0	0.974	0.941–0.989	0.95	**<0.001**	0.2	0.6
	C3-DC (C4-OH)	84.8	0.650	0.328–0.836	0.59	**0.003**	0.7	0.8
	C4	100.0	0.904	0.772–0.960	0.94	**<0.001**	0.02	0.3
	C5	100.0	0.867	0.715–0.941	0.83	**<0.001**	0.3	0.6
	C5-OH (C3-DC-M)	97.8	0.653	0.344–0.836	0.58	**0.004**	0.3	0.6
	C5:1	89.1	0.085	0.259–0.344	−0.10	0.639	0.9	0.9
	C10:2	89.1	0.350	−0.057-0.659	0.36	0.095	0.3	0.6
Aminoacids	Ala	100.0	0.941	0.840–0.976	0.88	**<0.001**	0.04	0.5
	Arg	100.0	0.855	0.692–0.935	0.81	**<0.001**	0.4	0.6
	Asn	100.0	0.870	0.719–0.943	0.87	**<0.001**	0.2	0.6
	Asp	100.0	0.750	0.432–0.893	0.77	**<0.001**	0.01	0.2
	Cit	100.0	0.958	0.905–0.982	0.96	**<0.001**	0.8	0.9
	Gln	100.0	0.709	0.432–0.865	0.60	**0.003**	0.3	0.6
	Glu	100.0	0.896	0.694–0.960	0.95	**<0.001**	0.006	0.2
	Gly	100.0	0.928	0.836–0.969	0.89	**<0.001**	0.1	0.6
	His	100.0	0.784	0.562–0.902	0.68	**<0.001**	0.3	0.6
	Ile	100.0	0.910	0.803–0.961	0.88	**<0.001**	0.3	0.6
	Leu	100.0	0.806	0.600–0.912	0.78	**<0.001**	0.3	0.6
	Lys	100.0	0.475	0.108–0.733	0.45	**0.03**	0.1	0.6
	Met	100.0	0.653	0.344–0.835	0.56	**0.006**	0.5	0.6
	Orn	100.0	0.901	0.785–0.957	0.93	**<0.001**	0.4	0.6
	Phe	100.0	0.648	0.339–0.832	0.42	**0.05**	0.5	0.6
	Pro	100.0	0.953	0.889–0.980	0.89	**<0.001**	0.1	0.6
	Ser	100.0	0.812	0.607–0.916	0.87	**<0.001**	0.5	0.7
	Thr	100.0	0.926	0.836–0.968	0.93	**<0.001**	0.4	0.6
	Trp	100.0	0.548	0.195–0.778	0.57	**0.004**	0.6	0.7
	Tyr	100.0	0.519	0.159–0.760	0.62	**0.002**	0.3	0.6
	Val	100.0	0.790	0.571–0.905	0.73	**<0.001**	0.4	0.6
Biogenic Amines	ADMA	100.0	0.921	0.824–0.965	0.87	**<0.001**	0.6	0.7
	alpha-AAA	100.0	0.688	0.400–0.853	0.60	**0.003**	0.2	0.6
	Carnosine	100.0	0.976	0.944–0.990	0.92	**<0.001**	0.4	0.6
	Creatinine	100.0	0.987	0.970–0.994	0.97	**<0.001**	0.7	0.8
	Kynurenine	100.0	0.744	0.487–0.882	0.80	**<0.001**	0.3	0.6
	Met-SO	100.0	0.651	0.329–0.836	0.78	**<0.001**	0.4	0.6
	Putrescine	100.0	0.895	0.772–0.954	0.88	**<0.001**	0.3	0.6
	SDMA	100.0	0.964	0.917–0.984	0.97	**<0.001**	1.0	1.0
	t4-OH-Pro	100.0	0.972	0.937–0.988	0.93	**<0.001**	0.2	0.6
	Taurine	100.0	0.861	0.478–0.952	0.89	**<0.001**	0.001	0.04
Glycerophospholipids	PC aa C32:0	97.8	0.770	0.531–0.896	0.77	**<0.001**	0.99	1.0
	PC aa C32:1	80.4	0.763	0.515–0.892	0.77	**<0.001**	0.97	1.0
	PC aa C32:3	71.7	0.154	0.126–0.280	−0.11	0.6	0.7	0.8
	PC aa C34:1	100.0	0.778	0.542–0.900	0.75	**<0.001**	0.2	0.6
	PC aa C34:2	87.0	0.576	0.218–0.796	0.54	**0.007**	0.09	0.6
	PC aa C36:1	100.0	0.828	0.634–0.923	0.75	**<0.001**	0.4	0.6
	PC aa C36:3	76.1	0.623	0.287–0.821	0.60	**0.003**	0.05	0.5
	PC aa C36:4	95.7	0.342	−0.087-0.660	0.52	**0.01**	0.2	0.6
	PC aa C38:3	89.1	0.602	0.256–0.810	0.52	**0.010**	0.5	0.6
	PC aa C38:4	73.9	0.631	0.298–0.826	0.61	**0.002**	0.4	0.6
	PC aa C38:5	73.9	0.510	0.124–0.760	0.65	**0.001**	0.1	0.6
	PC aa C38:6	73.9	0.466	0.065–0.735	0.63	**0.001**	0.06	0.5
	PC aa C40:2	76.1	0.053	−0.359-0.448	−0.001	>0.9	0.6	0.7
	PC aa C40:5	76.1	0.680	0.376–0.851	0.36	0.09	0.7	0.9
	PC aa C40:6	82.6	0.669	0.357–0.846	0.70	**<0.001**	0.3	0.6
	PC ae C34:1	97.8	0.853	0.683–0.935	0.50	**0.02**	0.8	0.9
	PC ae C36:3	89.1	0.640	0.312–0.831	0.43	**0.04**	0.8	0.9
	PC ae C36:4	73.9	0.820	0.626–0.919	0.44	**0.04**	0.2	0.6
	PC ae C36:5	71.7	0.773	0.534–0.897	0.55	**0.006**	0.3	0.6
	PC ae C38:4	76.1	0.759	0.513–0.890	0.59	**0.003**	0.08	0.6
	PC ae C40:5	91.3	0.681	0.389–0.850	0.48	**0.02**	0.4	0.6
Sphingolipids	SM C16:0	73.9	0.804	0.590–0.912	0.68	**0.001**	0.07	0.6
	SM C16:1	97.8	0.780	0.545–0.900	0.57	**0.004**	0.4	0.6
	SM C18:0	71.7	0.760	0.511–0.891	0.63	**0.001**	0.3	0.6
	SM C18:1	95.7	0.852	0.686–0.934	0.66	**0.001**	0.3	0.6
	SM C24:1	71.7	0.814	0.608–0.917	0.75	**<0.001**	0.2	0.6
Sugars	H1	100.0	0.971	0.934–0.988	0.89	**<0.001**	0.6	0.7

^a^ The full list of individual metabolites is available at https://biocrates.com/wp-content/uploads/2022/02/biocrates-p180-list-of-metabolites-v2-2021.pdf (accessed 30 October 2022).

^b^ *p* < 0.05 in bold.

LOD, limit of detection; ICC, interclass correlation coefficient; CI, confidence interval; r rho Spearman correlation coefficient.

We investigated the following clinical factors for association with these five metabolites: age, sex, body mass index (BMI), and axial length (AXL) (Table 4). Only BMI was found to be associated with C5:1 (*p* = 0.008).

**TABLE 4:** Effect of age, sex, BMI, and AXL on interocular differences (right eye–left eye) on selected metabolites.

Characteristics	C5: 1^a^		C10: 2		PC aa C32: 3		PC aa C40: 2		PC aa C40: 5	
	β (SE)	p^b^	β (SE)	p	β (SE)	p	β (SE)	p	β (SE)	p
Age, years	0.0003 (0.0004)	0.5	−0.0003 (0.0003)	0.2	−0.0001 (0.0001)	0.2	0.00001 (0.00004)	0.9	0.0001 (0.0003)	0.9
Sex, female	0.002 (0.006)	0.7	−0.0003 (0.004)	0.9	0.001 (0.001)	0.3	−0.001 (0.001)	0.3	−0.002 (0.003)	0.6
BMI	**−0.001 (0.001)**	**0.008^b^**	−0.0003 (0.0003)	0.4	0.0001 (0.0001)	0.2	−0.00001 (0.0001)	0.9	−0.0001 (0.0003)	0.7
AXL, right eye, mm	−0.003 (0.003)	0.3	−0.002 (0.002)	0.3	−0.0002 (0.0004)	0.6	−0.00002 (0.0003)	0.9	0.001 (0.002)	0.5
AXL, left eye, mm	−0.001 (0.003)	0.6	−0.0009 (0.002)	0.6	0.00001 (0.0003)	1.0	−0.0001 (0.0002)	0.6	0.001 (0.001)	0.4

Linear regression analysis. *β* beta coefficient, SE, standard error; BMI, body mass index; AXL, axial length.

^a^ The full list of individual metabolites is available at https://biocrates.com/wp-content/uploads/2022/02/biocrates-p180-list-of-metabolites-v2-2021.pdf (accessed 30 October 2022).

^b^ *p* < 0.05 in bold.

**FIGURE 1:**
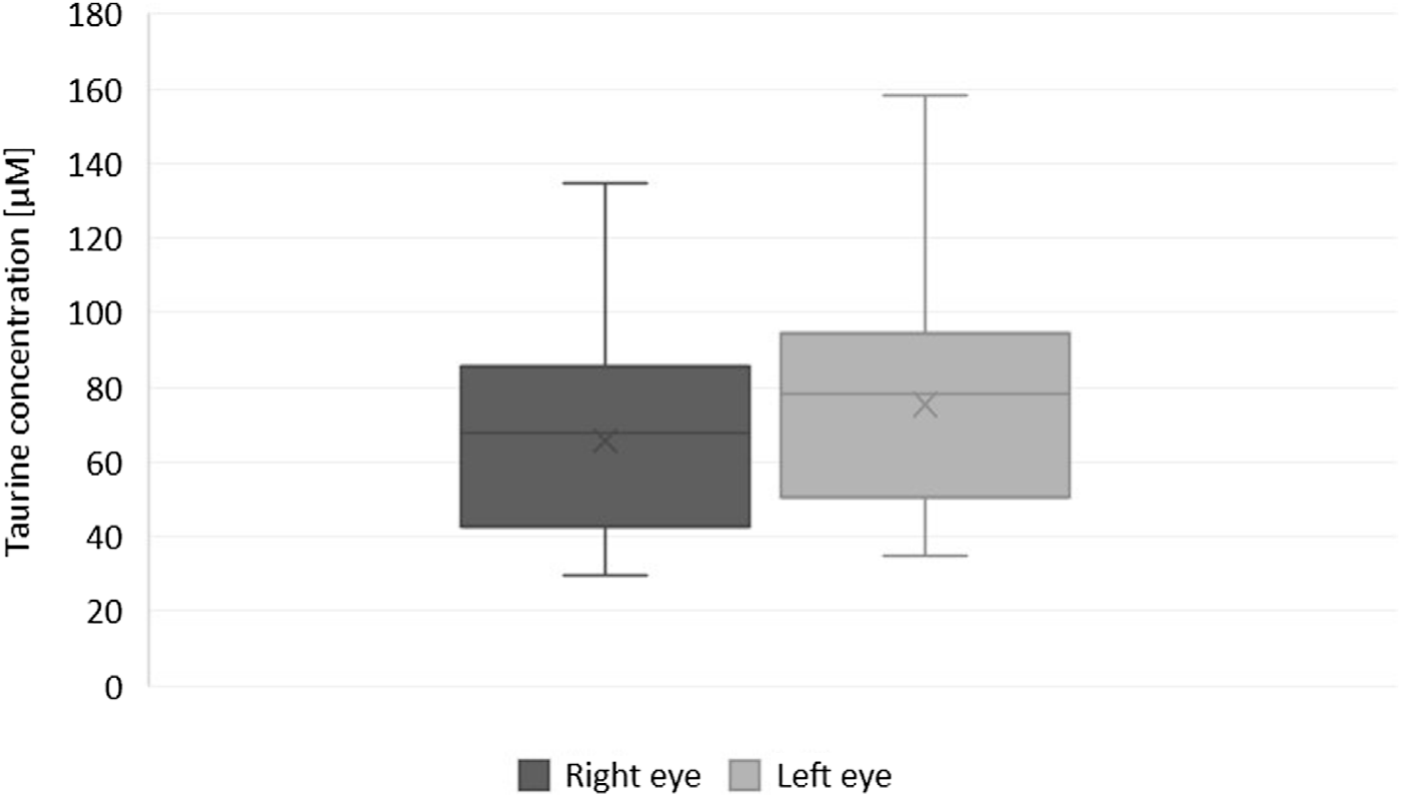
Box and whisker plot showing differences in the concentration of taurine in fellow eyes (Wilcoxon test with Benjamini–Hochberg correction for multiple comparisons, *p* = 0.037).

**FIGURE 2:**
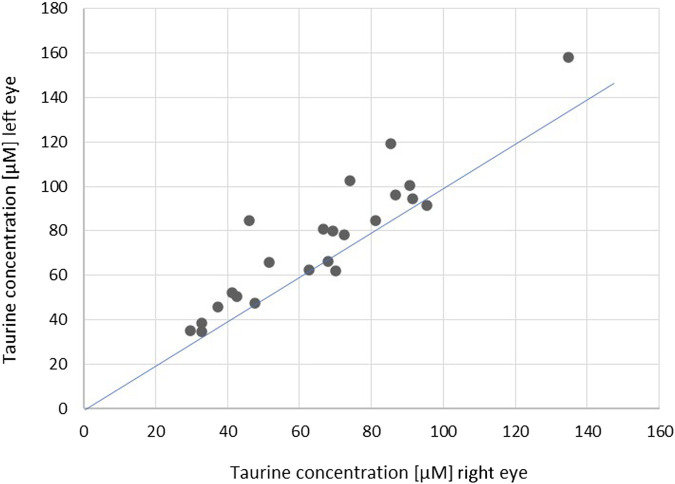
Scatterplot showing correlations of the concentration of taurine in fellow eyes (r = 0.89, *p* < 0.001; ICC = 0.861 [0.478–0.952]). The blue line indicates the theoretical perfect interocular agreement of concentrations.

Although the taurine concentration differed significantly between fellow eyes and was found lower in the left eye of only four patients (Figures 1, 2), there was a linear relation (as visualized in Figure 2) with a good ICC (0.861 CI_95_ [0.478–0.952]) and strong correlation (r = 0.89, *p* < 0.001).

## 4 Discussion

The partial anatomical asymmetry of both eyes has been described in the literature (Li et al., 2013; Hwang et al., 2014; Jacobsen et al., 2015; Yang et al., 2016; Cameron et al., 2017; Linderman et al., 2018; Baniasadi et al., 2020; Jiang et al., 2021; Kim et al., 2021; Albarrán-Diego et al., 2022; Lu et al., 2022; Song and Hwang, 2022). We investigated whether this extrapolates to the AH metabolomic composition. The aim of the study was to determine whether concentrations of various metabolites in one eye are representative of the fellow eye of the same patient.

In general, there were minimal interocular differences and substantial interocular correlation in most of the studied metabolites. However, we found some differences between both eyes. We demonstrated interocular asymmetry, though with high correlation, for concentrations of taurine. Taurine is an aminosulfonic acid that plays a role in several intracellular biological processes, including osmoregulation, antioxidation, and retinal development (Ripps and Shen, 2012). The retina is the most taurine-rich organ in the body, containing more taurine than any other amino acid (Castelli et al., 2021). People following vegetarian or vegan diets have been shown to have lower levels of taurine in their plasma (Laidlaw et al., 1988). However, this would not explain the interocular difference.

On the other hand, correlations were not significant but the concentrations were comparable for two acylcarnitines (C5: 1 and C10: 2) and three glycerophospholipids (PC aa C32: 3, PC aa C40: 2, and PC aa C40: 5). Using regression analysis, we tested their association with clinical factors (age, sex, BMI, and AXL). Except for the association of C5: 1 with BMI, no other relations were confirmed.

The general role of acylcarnitines is to transport acyl groups (organic acids and fatty acids) from the cytoplasm into the mitochondria so they can be broken down to produce energy. This process is known as beta-oxidation. C5: 1 is a member of the most abundant group of carnitines in the body, comprising more than 50% of all acylcarnitines quantified in tissues and biofluids (Makarova et al., 2019). Glycerophospholipids (including phosphatidylcholine) are the main component of biological membranes.

We speculate that the asymmetry of these six metabolites could be due to different rates of either production or metabolism. In contrast to this locally based hypothesis, the asymmetry in the anatomy is worth noting. Lu et al., who assessed the asymmetry of choroidal thickness, suspected that it can be attributed to asymmetrical choroidal blood flow. This may result from an anatomical asymmetry of the aortic arch and common carotid arteries, as well as from the variable anatomy of ciliary arteries and choroidal venous drainage (Lu et al., 2022). In case of two acylcarnitines and two PCs coefficient of variation calculated for the QC2 samples was higher than observed between-eyes variability (Table 2), therefore, observed variations can partly be affected by the analytical variability.

According to Cameron et al., researchers should avoid the potential pitfall of forcing an expectation of symmetry on paired structures, and clinicians should be aware of both the benefits and limitations in extrapolating single-eye data to the fellow eye in diagnosis and prognosis (Cameron et al., 2017). Based on our results, we confirm that the assumption of metabolomic symmetry of AH composition in fellow eyes should be approached with caution, as there may be asymmetry in the case of certain metabolites/metabolite categories. It is worth mentioning, that we did not observe the relationship between inter-individual variability in metabolites’ concentrations and between-eyes differences in the concentrations (data not shown). Our findings may have clinical significance when planning studies and interpreting results.

The main limitation of the study is the relatively low number of participants, which were all from a single center. The use of a single ethnicity minimizes the confounding effects of ethnicity but impacts the generalizability of the results. Furthermore, only selected groups of metabolites were analyzed in the study. Due to the cross-sectional design, patients with ocular disease at a very early stage or those who will later develop ocular diseases may have been included. However, as opposed to the relatively easy collection of, e.g., blood or urine, AH can only be obtained surgically in low amounts of about 0.1 mL. Therefore, longitudinal studies are rather infeasible. Additionally, applied methodology allows measurement of only 188 specific metabolites. Untargeted metabolomics may reveal intraocular differences in other metabolites, not included in the kit used. Another limitation is the lack of information on the Lens Opacities Classification System (LOCS) grading. It would be helpful for the interpretation of our results, e.g., taurine levels. There is only the information regarding the opacified layer of the lens but with interindividual variability (cortical/nuclear/subcapsular, Supplementary Table S1. Baseline characteristics of patients). However, on the slit lamp examination the cataracts were symmetrical within the same individuals.

On the other hand applied technology allows quantification of metabolites, which would not be possible using untargeted approach. Another strength of the study is the simultaneous analysis of a relatively high number of metabolites from different groups. Furthermore, the simultaneous collection of AH from fellow eyes is a rather unique situation. Samples obtained in such a way would make it possible to minimize the between-group impacts of genetic, dietary, lifestyle, systemic, or environmental factors, as their influence is presumably comparable on both eyes (e.g., age, BMI, sex, smoking, diet, circadian rhythms, time of year, concomitant systemic diseases, and medication) (Crews et al., 2009).

Our results may serve as a basis for future studies. An interesting direction of future research would be the analysis of intraindividual biological variability of AH in time. As the acquisition of AH is surgical, patients with bilateral delayed (not simultaneous) cataract surgery could be included. The knowledge of results from the current study would be helpful to properly interpret such findings. Our results require validation in a different cohort and for different groups of metabolites. Variable analytical methodology using a multiplatform approach would be valuable for this purpose. In addition, the correlation of metabolite concentrations in serum and AH might help in identifying metabolites whose concentrations are more locally dependent. The intraindividual variability in the metabolome between eyes of different axial lengths would be informative as well as relation to the LOCS grading.

## 5 Conclusion

On the basis of our findings, we conclude that one eye is representative of the fellow eye in terms of the concentration of most of the analyzed metabolites, with only a few exceptions.

The degree of intraindividual variability in the AH of fellow eyes differs within particular metabolites/metabolite categories.

## Data Availability

The raw data supporting the conclusion of this article will be made available by the authors, without undue reservation.
