# Supplementary material for: Extent of interocular (a)symmetry based on the metabolomic profile of human aqueous humor
Source: Front Mol Biosci. 2023 Mar 29;10:1166182. doi: 10.3389/fmolb.2023.1166182 (PMC10090416; doi:10.3389/fmolb.2023.1166182)
Supplement: Supplementary file 1 [file Table1.DOCX]

Supplementary Material

**Extent of interocular (a)symmetry based on the metabolomic profile of human aqueous humor**

**Karolina Pietrowska^1†^, Diana Anna Dmuchowska^2*†^, Adrian Godlewski^1^, Emil Tomasz Grochowski^2^, Małgorzata Wojnar^2^, Wioleta Gosk^1^, Joanna Konopinska^2^, Adam Kretowski^1,3^ and Michal Ciborowski^1*^**

^1^Clinical Research Center, Medical University of Bialystok, Bialystok, Poland

^2^Department of Ophthalmology, Medical University of Bialystok, Bialystok, Poland

^3^Department of Endocrinology, Diabetology and Internal Medicine, Medical University of Bialystok, Bialystok, Poland

**†**These authors contributed equally to this work

*** Correspondence:**

Dr. Michal Ciborowski, Metabolomics Laboratory, Clinical Research Center, Medical University of Bialystok, Poland, michal.ciborowski@umb.edu.pl

or Dr. Diana Anna Dmuchowska, Department of Ophthalmology, Medical University of Bialystok, Bialystok, Poland, [diana.dmuchowska@umb.edu.pl](mailto:diana.dmuchowska@umb.edu.pl)

Supplementary Table S1.Baseline characteristics of patients

| Number | Age (years) | Gender | BMI | Type of cataract | Comorbidities | Other medications |
| --- | --- | --- | --- | --- | --- | --- |
| 1 | 41 | Female | 20,4 | Nuclear/Cortical | none | none |
| 2 | 90 | Male | 31,1 | Nuclear/Cortical | Benign Prostatic Hyperplasia | none |
| 3 | 74 | Female | 27,9 | Cortical | Atrial Fibrillation, Osteoarthritis | Bisoprololi fumaras, Dabigatranum etexilatum, Rosuvastatinum, Candesartanum cilexetilum, Clonidini hydrochloridum, Spironolactonum |
| 4 | 73 | Male | 30,9 | Nuclear/Subcapsular | Atrial Fibrillation, Hypertension, Gout | Lercanidipini hydrochloridum, Telmisartanum, Metoprololum, Furosemidum,Allopurinolum, Acenocoumarolum |
| 5 | 80 | Male | 21,4 | Nuclear/Subcapsular | Multiple myeloma | Fentanylum, Acidum acetylsalicylicum, Cyclophosphamidum, Dexamethasonum, Esomeprazolum |
| 6 | 76 | Female | 28,8 | Nuclear/Cortical | Atrial Fibrillation , history of Stroke, Atherosclerosis, Dyslipidemia | Perindoprilum argininum, Bisoprololi fumaras, Dabigatranum etexilatum, Piracetamum, Rosuvastatinum |
| 7 | 71 | Female | 33,1 | Nuclear/Subcapsular | Diabetes Mellitus, Coronary Artery Disease, Hypertension, Hyperlipidemia, Atherosclerosis, Osteoarthritis, biological aortic valve replacement | Insulinum humanum, Metformini hydrochloridum, Warfarinum, Perindoprilum argininum, Bisoprololi fumaras, Fenofibratum, Atorvastatinum, Empagliflozinum + Metforminum |
| 8 | 89 | Male | 25,7 | Nuclear | Hypertension, Benign Prostatic Hyperplasia | Finasteridum, Escitalopramum, Acidum folicum, Nebivololum, Tamsulosinum, Dexlansoprazolum |
| 9 | 70 | Female | 30,1 | Nuclear | Hypertension | Bisoprololi fumaras, Lisinoprilum + Amlodipinum, Dexlansoprazolum, Acidum acetylsalicylicum + Glycinum |
| 10 | 88 | Female | 37,9 | Nuclear/Cortical | Chronic Obstructive Pulmonary Disease, Venous Thrombosis | Rivaroxabanum |
| 11 | 46 | Female | 16,4 | Nuclear/Subcapsular | Coronary Artery Disease, Paroxysmal Atrial Fibrillation, Gastroesophageal Reflux Disease | Metoprololi tartras, Esomeprazolum |
| 12 | 84 | Female | 30,8 | Nuclear/Cortical/Subcapsular | Chronic Obstructive Pulmonary Disease | none |
| 13 | 81 | Male | 29,7 | Subcapsular | Diabetes Mellitus, Coronary Artery Disease, Hypertension | Pentoxifyllinum, Rivaroxabanum, Doxazosinum, Epleronum, Simvastatinum, Bencyclani fumaras, Torasemidum |
| 14 | 73 | Male | 22,0 | Nuclear | Chronic Obstructive Pulmonary Disease | Salbutamolum, Formoterolum |
| 15 | 84 | Male | 26,1 | Nuclear/Cortical/Subcapsular | Coronary Artery Disease, Hypertension | Perindoprilum argininum, Nebivololum, Finasteridum, Torasemidum, Acidum acetylsalicylicum |
| 16 | 85 | Male | 27,2 | Nuclear/Cortical | Hypertension | Betahistini dihydrochloridum, Valsartanum |
| 17 | 75 | Female | 22,4 | Nuclear/Cortical | Hypertension, Dyslipidemia | Ramiprilum + Hydrochlorothiazidum, Amlodipinum, Metoprololi tartras, Atorvastatinum, Fenofibratum |
| 18 | 78 | Female | 31,6 | Nuclear | Diabetes Mellitus, Coronary Artery Disease, Hypertension, Parkinson's Disease, Hypothyroidism, history of Stroke | Metformini hydrochloridum, Gliclazidum, Amantadine sulfate, Levothyroxinum natricum, Atorvastatinum, Aceclofenacum, Vinpocetinum |
| 19 | 80 | Male | 35,6 | Nuclear/Cortical | Osteoarthritis, Hypertension, Gout | Enalaprili maleas, Ketoprofenum, Pantoprazolum, Nitrendipinum, Simvastatinum |
| 20 | 75 | Female | 28,0 | Nuclear/Cortical | Hypertension, Dyslipidemia, Atherosclerosis | Rosuvastatinum, Enalaprili maleas, Pentoxifyllinum, Bisoprololi fumaras, Bencyclani fumaras, Vinpocetinum, Esomeprazolum, Acidum acetylsalicylicum |
| 21 | 77 | Female | 22,7 | Nuclear/Cortical | Diabetes Mellitus, Hypertension | Acarbosum, Metformini hydrochloridum, Valsartanum |
| 22 | 66 | Female | 37,1 | Nuclear | Recurrent migraines, Deppressive Disorders | Levothyroxinum natricum, Rosuvastatinum, Citalopramum, Flunarizinum |
| 23 | 74 | Male | 26,9 | Nuclear/Cortical/Subcapsular | Benign Prostatic Hyperplasia, Atrial and Mitral Regurgitation, Kidney Stones, Hemorrhoids, Stupor, Personality and Behavioral Disorders | Memantine hydrochloride, Donepezili hydrochloridum |
